# Trochanteric stabilizing plate in the treatment of trochanteric fractures: a scoping review

**DOI:** 10.1080/17453674.2021.1954305

**Published:** 2021-07-23

**Authors:** Carl Erik Alm, Jan-Erik Gjertsen, Trude Basso, Kjell Matre, Stephan Rörhl, Jan Erik Madsen, Frede Frihagen

**Affiliations:** a Division of Orthopaedic Surgery, Oslo University Hospital, Oslo;; b Institute of Clinical Medicine, Faculty of Medicine, University of Oslo, Oslo;; c Department of Clinical Medicine, University of Bergen, Bergen;; d Department of Orthopaedic Surgery, St Olav’s University Hospital, Trondheim;; e Department of Orthopaedic Surgery, Østfold Hospital Trust, Grålum, Norway.

## Abstract

Background and purpose — The trochanteric stabilizing plate (TSP) may be used as an adjunct to a sliding hip screw (SHS) in the treatment of trochanteric fractures to increase construct stability. We performed a scoping review of the literature to clarify when and how the TSP may be useful.

Methods — A systematic search was performed in 5 databases and followed by a backwards-and-forwards citation search of the identified papers. 24 studies were included.

Results — 6 biomechanical studies and 18 clinical studies were included in the review. The studies presented mainly low-level evidence. All studies were on unstable trochanteric fractures or fracture models. Due to the heterogeneity of methods and reporting, we were not able to perform a meta-analysis. In the biomechanical trials, the TSP appeared to increase stability compared with SHS alone, up to a level comparable with intramedullary nails (IMNs). We identified 1,091 clinical cases in the literature where a TSP had been used. There were 82 (8%) reoperations. The rate of complications and reoperations for SHS plus TSP was similar to previous reports on SHS alone and IMN. It was not possible to conclude whether the TSP gave better clinical results, when compared with either SHS alone or with IMN.

Interpretation — The heterogeneity of methods and reporting precluded any clear recommendations on when to use the TSP, or if it should be used at all.

Internal fixation of trochanteric femoral fractures is usually performed with a plate or nail with a lag screw allowing axial compression to enhance fracture healing. The agreement between surgeons on implant choice is fair (Mellema et al. 2021). While a sliding hip screw (SHS) seems sufficient in stable trochanteric fractures (Parker and Handoll 2010), several guidelines recommend the use of intramedullary nails (IMNs) in more unstable fracture patterns (NICE 2011, Roberts and Brox 2015). Fixation without a lag screw is not recommended (Parker and Handoll 2010, Parker et al. 2018). Fractures involving the lateral wall, or with posteromedial comminution, are considered as unstable. This might cause excessive medialization of the femoral shaft, malunion, poor functional results, and even fixation failure (Parker 1996, Bretherton and Parker 2016). In addition, a thin lateral wall or a concomitant fracture through the greater trochanter increases the risk for an intra- or postoperative lateral wall fracture (Palm et al. 2007, Hsu et al. 2013). Under these circumstances, with a compromised lateral buttress, implant-preventing secondary displacement is required.

The trochanteric stabilizing plate (TSP) was introduced in the early 1990s as an adjunct to the sliding hip screw. The plate acts by buttressing the lateral trochanteric wall and is intended to prevent medialization of the femoral shaft (Figure 1). Despite being sparsely discussed in the literature, an SHS with an additional TSP has been widely used in some countries and regions for decades (Lunsjö et al. 2001, Bong et al. 2004, Gupta et al. 2010, Knobe et al. 2013, Hsu et al. 2015, Alm et al. 2021). We reviewed the literature on TSP to clarify existing evidence and aid in the decision-making on when to use a TSP.

**Figure 1.**
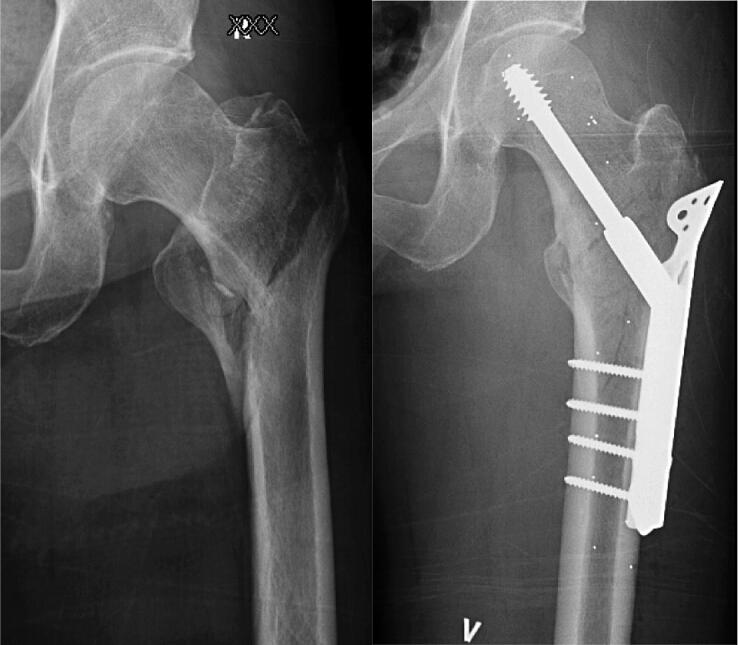
Pre- and postoperative images of a AO/OTA 31-A2 fracture operated on with a sliding hip screw with trochanteric stabilizing plate (TSP). The TSP should prevent excessive medialization of the femoral shaft by buttressing the lateral trochanteric wall. In this case, a loss of medial buttress with a large lesser trochanter fragment and a thin lateral wall would strengthen the traditional indication for a TSP.

## Method

We applied the recommendations from the Cochrane collaboration (Higgins et al. 2020) and the methodological framework for scoping reviews as proposed by Arksey and O’Malley (2005).

### Research questions

1. What are the mechanical properties of the SHS plus TSP compared with SHS alone or intramedullary implants?
2. Does the TSP lead to an improved clinical outcome compared with SHS alone or IMN?
3. How does the TSP function in terms of non-union, mechanical failure, and reoperations?
4. Is it possible to establish guidelines for TSP use based on the existing evidence?

### Eligibility criteria

All papers, both clinical and biomechanical, reporting outcomes related to TSP use in trochanteric fracture treatment were included in the review. We excluded studies reporting 3 cases or less, or where the TSP was used for indications other than acute trochanteric fractures.

### Information sources

**Figure 2.**
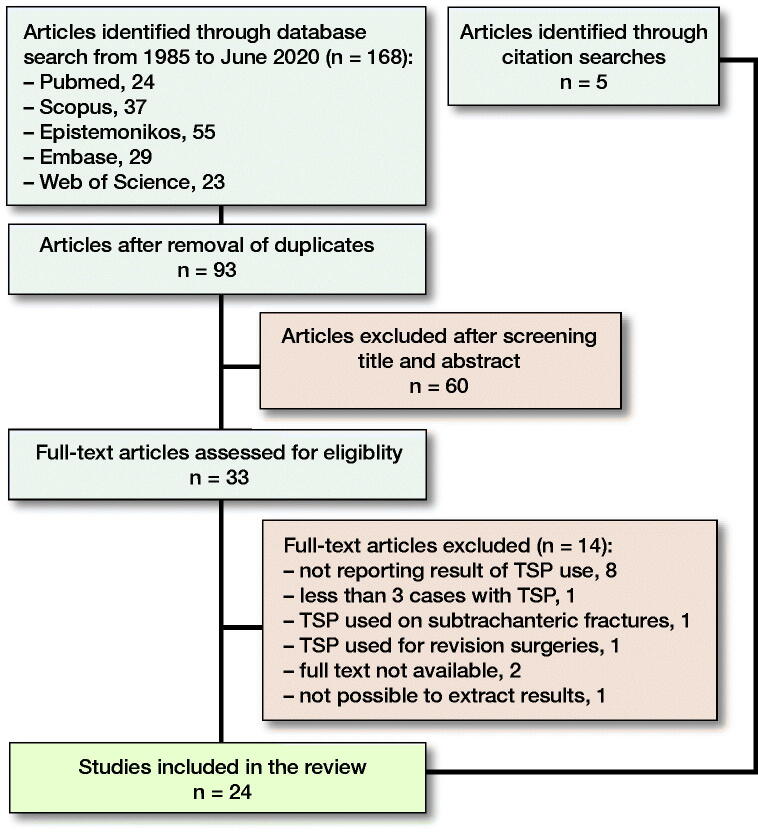
Flow chart of papers in the review. The papers identified were from Norway, Sweden, Switzerland, Germany, Taiwan, India, United States, Canada, Northern Ireland, South Korea, and Egypt. Search strategy: Languages: All. Search terms/-strings: Title/Abstract (“trochanteric stabilising plate” OR “trochanteric stabilizing plate” OR “trochanteric stabilisation plate” OR “trochanteric stabilization plate” OR “lateral support plate” OR “trochanter stabilising plate” OR “trochanter stabilizing plate” OR “trochanter stabilization plate” OR “trochanter stabilisation plate”).

A systematic search through PubMed, Scopus, Web of Science, Embase, and Epistemonikos was performed and last updated on June 25, 2020 by the 1st author (CEA). The complete search strategy is shown in the legend to Figure 2. In addition, we did a backwards search of all references of the papers identified and a forward search of papers citing the identified publications. We also manually searched the reference lists of review papers, meta-analyses, and guidelines until no new papers turned up.

### Study selection

Irrelevant studies were excluded based on title and abstract screening. Full text versions of the remaining studies were screened for eligibility by CEA and FF.

### Funding and potential conflicts of interest

No funding was received. The authors declare no conflicts of interest.

## Results

### Study selection

After removal of duplicates, 93 unique papers were identified for further analysis (Figure 2). Based on screening of title and/or abstract, 60 records were excluded, leaving 33 studies for full text evaluation. Of these 33 studies, 14 were excluded for various reasons (Figure 2). The citation searches identified 5 additional papers (Babst et al. 1993, David et al. 1996, Friedl and Clausen 2001, Klinger et al. 2005, Bonnaire et al. 2007).

### Summary of the literature

6 biomechanical studies (Table 1, see Supplementary data) and 18 clinical studies (Tables 2 and 3, see Supplementary data) were identified. The clinical studies all reported surgical outcomes, and all but 2 reported at least 1 relevant clinical outcome. All papers studied unstable trochanteric fractures or fracture models.

**Table 1.** Included biomechanical studies reporting on sliding hip screw (SHS) with trochanteric support plate (TSP), compared to either intramedullary nail (IMN, 5 studies) or SHS alone (1 study), and 95° angled blade plate (1 study)

Reference	Specimen (n)	TSP (n)	Comparator	Fracture model	Outcome
Gцtze et al. 1998	Plastic (32), Cadaver (24)	4	IMN, 95°- blade plate	AO 31 A2, A3	Significant higher load to failure with both types of IMN compared with SHS plus TSP
Friedl and Clausen 2001	Plastic (8), Cadaver (2)	5	IMN	AO 31 A2, A3, Subtrochanteric	Higher total and earlier deformation with SHS plus TSP compared with IMN during cyclic loading
Su et al. 2003	Cadaver (10)	10	SHS	AO 31 A3	Significant less sliding distance and displacement of the femoral head in the TSP group
Bong et al. 2004	Cadaver (6)	6	IMN	Evans-Jensen 5	No sign differences in displacement found between the 2 groups during static and cyclic loading
Bonnaire et al. 2007	Cadaver (32)	8	IMN	AO 31 A2.3	Cutout dependant on BMD. All implants sufficient as long as BMD > 0.6 g/cm^3^
Walmsley et al. 2016	Composite (24)	NA	IMN	AO 31 A3	Similar stiffness but reduced strength with SHS compared with an IMN.

BMD = bone mineral density

**Table 2.** Methods of the clinical studies included reporting on the use of sliding hip screw (SHS) with trochanteric support plate (TSP), without comparator (8 studies) or comparing with intramedullary nail (IMN, 6 studies), SHS alone (5 studies), proximal femur locking plate (PFLP, 1 study), Medoff sliding plate (MSP, 1 study) and dynamic condylar screw (DCS, 1 study). In one study an anti-rotation screw (ARS) was used as an addition to SHS

Reference	Design	TSP (n)	Comparator	Fracture classification
Studies without comparator				
Babst et al. 1993	Retrospective cohort	17	None	AO A2,3, A3.3
Hoffmann et al. 1994	Retrospective cohort	19	None	AO A2, A3
David et al. 1996	Prospective cohort	22	None	AO A3
Babst et al. 1998	Retrospective cohort	46	None	AO A2.2, A2.3, A3.3
Gupta et al. 2010	Prospective cohort	46	None	AO
Cho et al. 2011	Retrospective cohort	27	None	AO A2
Prabhakar and Singh 2016	Prospective cohort	25	None	AO A2.1, A2.2, A2.3
Shetty et al. 2016	Prospective cohort	32	None	Evans-Jensen 2–3
Studies with comparator				
Madsen et al. 1998	Retrospective cohort	85	SHS, IMN	Evans-Jensen 3–5
Lunsjц et al. 2001	Prospective cohort	49	MSP, DCS	Evans-Jensen 3–5
Nuber et al. 2003	Retrospective cohort	64	IMN	AO A2.2, A2.3
Klinger et al. 2005	Retrospective cohort	51	IMN	AO A2.3
Hsu et al. 2015	Retrospective cohort	46	SHS	AO A2.1, A2.2, A2.3
Tucker et al. 2018	National database/registry	158	SHS, IMN	AO A2.2, A2.3, A3
Haddon et al. 2019	RCT	50	SHS	Evans-Jensen 3–5
Mьller et al. 2019	Retrospective cohort	100	SHS + ARS, IMN	AO A2
Selim et al. 2020	RCT	20	PFLP	AO A2.2, A2.3
Fu et al. 2020	Retrospective cohort	234	IMN	AO A2, A3

**Table 3.** Results of the clinical studies included reporting on the use of sliding hip screw (SHS) with trochanteric support plate (TSP), without comparator (8 studies) or comparing with intramedullary nail (IMN, 6 studies), SHS alone (5 studies), proximal femur locking plate (PFLP, 1 study), or other extramedullary implants (1 study)

Reference	Clinical outcome	Mechanical outcome	Mechanical failure/non -union (n)	Reope-rations n (%)	“Authors’ conclusion”
**Studies without comparator**					
Babst et al. 1993	13 patients little or no pain. 10 patients unlimited walking distance.	6 patients 10–25 mm protrusion of lag screw.	0	5 (29)	The TSP prevents excessive laterali-zation of the greater trochanter
Hoffmann et al. 1994	12 patients walking distance > 200 m. 14 patients little or no pain.	5 patients 10–20 mm protrusion of lag screw. No reoperations at a mean of 6 months follow up. (2 died within 30 d)	0	0 (0)	Low rate of complications and good functional results. More difficult implementation than SHS alone
David et al. 1996	Good functional results dislocation	1 patient with varus	0	0 (0)	TSP recommended in treatment of AO A3 fractures
Babst et al. 1998	Good functional results. 87% excellent/good Salvati Wilson score	Mean impaction 9.5 mm. Mean shortening 6.8 mm.	3	6 (13)	The TSP effectively supports the greater trochanter when the lateral buttress is compromised
Gupta et al. 2010	Good functional results	All fractures healed. 2 TSP removals as buttress is compromised	2	2 (7)	TSP seems to be a useful device for lateral wall reconstruction
Cho et al. 2011	Good functional results with Parker and Palmer mobility score 6.2 (7.2 preop.)	All fractures healed. 1 excessive lag screw sliding and 1 lag screw breakage	0	0 (0)	Additional fixation enables stable fixation of trochanteric fractures and a high rate of union
Prabhakar and Singh 2015	85% excellent/good Harris Hip Score	2 patients with varus collapse and shortening > 2 cm	1	2 (8)	SHS plus TSP is a biomechanically stable construct that allows for lateral wall reconstruction
Shetty et al. 2016	19/32 excellent/good Harris Hip Score	High union rate. Mean RUSH score 21	0	0 (0)	Fixation of unstable trochanteric fractures with SHS plus TSP is an effective technique with good functional and radiological outcome
**Studies comparing SHS/TSP with SHS**					
Hsu et al. 2016	NA	Less lag screw sliding, postop. lateral wall fractures and reoperations with TSP	1	1 (2)	The TSP significantly decreases lag screw sliding distance and reoperation rate in A2 fractures with a critically thin lateral wall compared with SHS alone
Haddon et al. 2019	No difference in functional outcome measured with Merle d’Aubigne score	No difference in radiological outcome or reoperation rates	3	3 (6)	No certain beneficial effect of the TSP on unstable trochanteric fractures compared with SHS alone
**Studies comparing SHS/TSP with other extramedullary implants**					
Lunsjц et al. 2001	No difference in functional outcome	No difference in fixation failure/revisions	3	3 (6)	Extramedullary fixation yields good results with low rate of complications and good functional results. No difference between the examined implants
Selim et al. 2020	Better functional outcome and time to union with SHS	Fewer hardware failures and revisions in the SHS plus plus TSP TSP group	1	1 (5)	SHS plus TSP yields better results than the PFLP in trochanteric fracture treatment
**Studies comparing SHS/TSP with IMN**					
Nuber et al. 2003	Less pain with IMN	Similar complication rates	NA	6 (9)	IMN recommended over SHS plus TSP due to less pain in the IMN group at follow up after 6 months
Klinger et al. 2005	No difference in functional outcome measured with Merle d’Aubigne score	Fewer revisions with IMN. 17% vs 22%	NA	11 (22)	IMN recommended for unstable trochanteric fractures due to more complications in the SHS plus TSP group
Fu et al. 2020 (A2)	No difference in EQ-5D or functional status. More residual pain in the DHS with TSP group	No difference in healing, failure rate or rate of reoperations	10	6 (4)	Good surgical outcome with SHS plus TSP. Comparable results with IMN for both AO A2 on A3 fractures.
Fu et al. 2020 (A3)	No difference in EQ-5D or functional status. More residual pain in the DHS with TSP group	No difference in healing, failure rate or rate of reoperations	2	6 (9)	Good surgical outcome with SHS plus TSP. Comparable results with IMN for both AO A2 on A3 fractures
**Studies comparing SHS/TSP with both SHS and IMN**					
Mьller et al. 2019	NA	Better TAD, reduction, and lag screw positioning with IMN. More implant- related complications with SHS ± TSP	11	21 (21)	SHS with TSP associated with more complications and worse radiographi- cal results compared to IMN. IMN recommended for AO A2 fractures
Madsen et al. 1998	Trend towards better functional results with TSP	Less lag screw sliding with TSP. Similar complication rates	5	5 (6)	Fewer associated femoral shaft fractures with TSP compared to IMN and less medialization of the femoral shaft with TSP compared with SHS alone
Tucker et al. 2018	No difference in functional outcome after 12 months. Higher mortality rate with TSP	Similar complication rates	4	4 (3)	IMN conveys the best functional results and fewer revisions when compared with SHS alone or SHS with TSP

16 studies compared SHS plus TSP (SHS/TSP) with other implants. In 3 studies the comparator was SHS without TSP (Su et al. 2003, Hsu et al. 2015, Haddon et al. 2019). In 8 studies SHS/TSP was compared with IMN (Götze et al. 1998, Friedl and Clausen 2001, Nuber et al. 2003, Bong et al. 2004, Klinger et al. 2005, Bonnaire et al. 2007, Walmsley et al. 2016, Fu et al. 2020), and in 3 studies SHS/TSP were compared with both SHS and IMN (Madsen et al. 1998, Tucker et al. 2018, Müller et al. 2020). In addition, SHS/TSP was compared with other extramedullary implants in 2 papers (Lunsjö et al 2001, Selim et al. 2020). The lack of standardized methods and reporting of outcomes made a meta-analysis infeasible.

We identified 1,091 clinical cases in the literature where a TSP had been used as an adjunct to the SHS. Overall, 46 cases (4%) of mechanical failures and non-unions were reported. The number of reoperations was 82 (8%), including 19 routine removals of implants. The 10 prospective trials reported 15 reoperations (4%), while 67 (10%) reoperations were reported in the retrospective trials.

### Biomechanical studies

#### SHS/TSP versus SHS (Table 1, see Supplementary data)

Su et al. (2003) studied unstable trochanteric fracture models in 10 matched pairs of embalmed femora instrumented with an SHS with or without a TSP. The addition of the TSP to the SHS led to decreased displacement of the head fragment after cyclic loading at 750N.

#### SHS/TSP versus IMN (Table 1, see Supplementary data)

2 studies compared SHS/TSP with an IMN in both cadaveric and synthetic femora using various osteotomies. Friedl and Clausen (2001) concluded that the IMN was more resistant to deformation on cyclic loading than SHS with TSP. Götze et al. (1998) reported a higher load to failure with IMN than SHS with TSP. To compare the biomechanical properties of the SHS plus a TSP with an IMN, unstable, 4-part trochanteric fractures were created in 6 pairs of cadaveric human femora, matched by bone mineral density (BMD), by Bong et al. (2004). In their study the SHS plus TSP provided equal stability and similar ability to resist femoral shaft medialization as the IMN at 250–750 N loading. Walmsley et al. (2016) created unstable intertrochanteric fractures in 24 artificial femora showing similar stiffness but lower axial compression strength when SHS/TSP was compared with an IMN. Bonnaire et al. (2007) studied the influence of BMD on the risk of lag screw cut out in a trochanteric fracture model. They compared fixation with SHS/TSP with 2 types of IMN using cyclic loading at 2000N and found that if BMD was above 0.6 g/cm3 all implants provided sufficient stability to avoid fixation failure.

### Clinical studies

#### Studies reporting SHS/TSP without comparator (Tables 2 and 3, see Supplementary data)

We identified 8 patient series without comparator including from 17 to 46 patients, 234 in total. The TSP was mainly used in unstable fractures (Tables 3 and 4) (Babst et al. 1993, Hoffmann et al. 1994, David et al. 1996, Babst et al. 1998, Gupta et al. 2010, Cho et al. 2011, Prabhakar and Singh 2016, Shetty et al. 2016). The reporting of surgical and clinical results varied. All papers reported number of reoperations, and at least 1 radiographic and functional outcome. 4 papers reported results of a functional outcome score. All concluded that SHS plus TSP was a viable treatment option.

#### Studies comparing SHS plus TSP with SHS alone or with other extramedullary implants (Table 3, see Supplementary data)

SHS plus TSP was compared with SHS alone in 2 studies and with other extramedullary implants in 2 studies. In the only randomized trial in this review, 100 patients with unstable trochanteric fractures were randomized to SHS with or without a TSP. No clinically relevant differences between the groups were found, either in complications, secondary fracture displacement, or functional results (Haddon et al. 2019). Hsu et al. (2015) reported on 252 patients with AO/OTA 31 A2 trochanteric fractures. 205 patients were operated on with an SHS alone and 47 with SHS plus TSP. They performed a risk analysis for postoperative lateral wall fracture (LWF) and found that a lateral wall thickness (LWT) of less than 22 mm strongly predicted a postoperative fracture of the lateral wall. Further, they compared SHS alone (n = 125) and SHS plus TSP (n = 46) as treatment of fractures with a LWT < 22 mm and found that the TSP decreased lag screw sliding and reoperation rate. Lunsjö et al. (2001) performed a secondary analysis of a randomized trial with 569 patients with unstable trochanteric fractures. At the surgeon’s discretion 49 patients were operated on with an SHS and a TSP. No important difference was found between patients operated on with SHS and a TSP compared with patients operated with a Medoff plate or SHS without TSP. Selim et al. (2020) compared SHS plus TSP with a proximal femoral locking plate. The authors found better functional outcome, shorter time to union, and a lower failure rate in the SHS group.

#### Studies comparing SHS plus TSP with IMN (Table 3, see Supplementary data)

Nuber et al. (2003) compared SHS plus TSP with IMN in unstable trochanteric and subtrochanteric fractures and reported slightly better functional results and less pain in patients treated with an IMN. Complication rates and patient satisfaction were similar between the groups. Klinger et al. (2005) compared 51 patients treated with SHS/TSP with 122 patients treated with IMN. They found shorter operating time and hospital stay and fewer complications in the IMN group, but no differences in functional results. In the largest study (n = 234) included in the review, Fu et al. (2020) found no difference in functional scores, fracture healing, failure rate, or rate of reoperation when comparing SHS plus TSP with IMN in both AO type A2 and A3 fractures

#### Studies comparing SHS plus TSP with both SHS alone and with IMN (Table 3, see Supplementary data)

Madsen et al. (1998) compared a consecutive series of 85 patients with unstable trochanteric fractures treated with SHS plus TSP with 170 patients randomized to either an IMN or an SHS. They found a trend towards better functional results and less lag screw sliding in the TSP group, but an even distribution of complications. In a register-based study by Tucker et al. (2018) reporting on more than 3,000 fractures with IMN (598), SHS (2,474), and SHS plus TSP (158), a tendency towards fewer reoperations and better clinical results with IMN was found. Another retrospective cohort compared SHS with or without TSP and IMN (AO/OTA A2 fractures only) and reported a non-significant tendency toward fewer reoperations after IMN (Müller et al. 2020).

## Discussion

The identified studies presented mainly low-level evidence with only 1 prospective comparison and 1 relatively small randomized controlled trial. All studies reported on unstable fractures or fracture models. A meta-analysis was not possible due to the heterogeneity of the studies.

### Research question 1. What are the mechanical properties of the SHS plus TSP compared with SHS alone or with IMN?

The testing circumstances in the trials varied. 3 trials (Götze et al. 1998, Friedl and Clausen 2001, Walmsley et al. 2016) used supraphysiological loads, while in 2 trials (Su et al. 2003, Bong et al. 2004) the load applied was below normal loading associated with gait (Duda et al. 1997). In addition, the fracture models were simple, and thus not comparable to the comminution frequently seen in clinical practice. This complicates the interpretation of the results and limits the clinical value.

In 3 trials (Götze et al. 1998, Friedl and Clausen 2001, Walmsley et al. 2016) composite bones were used alone, or in combination with cadaver specimens. Synthetic bone is probably not adequate when testing a typically osteoporotic fracture model and the results may be of limited value, as the model does not mimic the bone loss predominant in hip fracture patients (Basso et al. 2014).

1 trial using cadaveric specimens found that SHS plus TSP provided sufficient stability within a clinical bone density range (Bonnaire et al. 2007).

The biomechanical studies using IMN as a comparator all showed that the TSP provided comparable stability to intramedullary nails (Table 2). The only biomechanical study comparing SHS with and without TSP (Su et al. 2003) used a highly unstable fracture model (AO/OTA A3) and found less displacement with an additional TSP. In comparison, the SHS alone has been reported to have less ability to withstand deformation after cyclic loading than IMN (Kaiser et al. 1997, Sommers et al. 2004).

Thus, the TSP appears to add stability to the osteosynthesis up to a level comparable with IMN.

### Research question 2. Does the TSP lead to an improved clinical outcome compared with SHS alone or with IMN?

The only randomized trial comparing SHS plus TSP with SHS alone was powered to detect a difference in lag screw sliding of 4 millimeters between SHS with and without TSP (Haddon et al. 2019). At 1-year follow-up a difference in lag screw sliding of less than 1 mm was found between the groups. With a broken lateral wall (n = 44) the difference was 3 mm in favor of the TSP group (not statistically significant). In the main clinical outcome measure in the trial, the Merle d’Aubigne-Postel score, a statistically non-significant 0.7 difference in favor of the group treated with SHS alone was found. The trial was not powered for subgroup analyses, but even with a larger number of patients included it is improbable that a meaningful clinical difference would have occurred.

A thin or fractured lateral wall may, however, be a predictor of mechanical failure (Palm et al. 2007, Hsu et al. 2015) and the TSP may have a beneficial effect under these circumstances as reported by Hsu et al. (2015).

A few studies (Madsen et al. 1998, Nuber et al. 2003, Klinger et al. 2005, Tucker et al. 2018, Müller et al. 2020) included in this review compared SHS plus TSP indirectly with IMN or SHS without TSP. From these studies it may be argued that the TSP protected against secondary fracture displacement. Madsen et al. observed a trend towards better functional results in the TSP group while the other publications failed to show any functional benefit of the TSP compared with IMN.

The findings above may be seen in light of 2 RCTs comparing SHS without TSP with IMN. Parker et al. (2017) included both stable and unstable fractures in a large trial. The authors reported slightly better regain of mobility in patients operated on with an IMN. Hardy et al. (1998) reported similar results in a randomized study of 100 patients. They explained their findings, at least in part, by the significantly larger lag screw sliding distance and subsequent limb shortening in the SHS group.

Based on the existing evidence it is not possible to conclude whether the TSP offers better clinical results than SHS alone, or when SHS plus TSP was compared with IMN for unstable trochanteric fractures.

### Research question 3. How does the TSP function in terms of non-union, mechanical failure, and reoperations?

A total of 1,091 SHS plus TSP were reported with 46 (4%) cases of healing problems and 82 (8%) reoperations for any cause. The 2010 Cochrane review (Parker and Handoll 2010), also including a high number of stable fractures, reported a (4%) reoperation rate and 3–4% healing complications and failures after SHS. In the same review the authors found an increased relative risk of cutout with the IMN, but a reduced risk of non-union (both statistically non-significant).

A study from the Norwegian Hip Fracture Register (Matre et al. 2013a) reported 10% reoperations at 3 years after AO/OTA A3 fractures and subtrochanteric fractures treated with SHS with or without TSP compared with 7% in the IMN group. This contrasts the findings in a randomized trial (Matre et al. 2013b) comparing IMN to SHS with or without TSP where a similar reoperation rate of 8% was reported after 12 months.

10 of the 19 included clinical trials were retrospective cohorts and chart reviews vulnerable to an under-reporting of serious complications and reoperations, as the patients may have sought advice elsewhere, or not at all (Table 2). 1 trial was from a register, equally prone to reporting minimum numbers of revision surgeries (Tucker et al. 2018). There was, however, no tendency to more reoperations in the prospective trials compared with the retrospective trials in our material.

The rate of complications and reoperations for SHS plus TSP was comparable to previous reports on trochanteric fractures treated with SHS alone or IMN.

### Research question 4. Is it possible to work out guidelines for TSP use based on the existing evidence?

All reports included in the review were on unstable fracture models or fractures. This implies that no authors believe that the TSP has a role in stable fractures (AO/OTA 31 A1 and Evans Jensen I–II). The results of the randomized trial comparing SHS with or without TSP suggest that the TSP has at best a limited role (Haddon et al. 2019). Some papers report, however, that the TSP increases stability compared with SHS alone (Su et al. 2003, Hsu et al. 2015) and with a similar stability to IMN (Madsen et al. 1998, Bong et al. 2004).

The limited literature identified, and the heterogeneity of methods and results, precludes any clear recommendations on when to use the TSP, or if it should be used at all. However, it might be argued that in practices where IMN is not available the TSP might be beneficial when treating trochanteric fractures with a thin or compromised lateral wall.

### Strengths and limitations

We believe that our literature search is exhaustive, and we have included both biomechanical and clinical trials. Some papers were not included due to insufficient reporting or failure to obtain a translation. A synthesis of functional results was not possible.

## Conclusion

This review did not identify literature clearly advising when to use a TSP. The findings indicated, however, that the TSP may provide a more stable construct, reducing lag screw sliding and medialization of the femoral shaft, than the SHS alone in unstable trochanteric fractures. Whether this translates into improved clinical outcomes compared with SHS alone or with IMN remains unclear. There is a need for high-quality, well-powered clinical trials with relevant outcome measures to clarify any role of the TSP in the treatment of trochanteric fractures.

## Supplementary Material

Supplemental Material

## Supplementary data

Tables 1–3 are available as supplementary data in the online version of this article, http://dx.doi.org/10.1080/17453674.2021.1954305
