# Supplementary material for: Trochanteric stabilizing plate in the treatment of trochanteric fractures: a scoping review
Source: Acta Orthop. 2021 Jul 23;92(6):733–8. doi: 10.1080/17453674.2021.1954305 (PMC8641671; doi:10.1080/17453674.2021.1954305)
Supplement: Supplemental Material [file IORT_A_1954305_SM1337.pdf]

Supplementary data

Table 1. Included biomechanical studies reporting on sliding hip screw (SHS) with trochanteric support plate (TSP), compared to either intramedullary nail (IMN, 5 studies) or SHS alone (1 study), and 95° angled blade plate (1 study)

Reference	Specimen (n)	TSP (n)	Comparator	Fracture model	Outcome
Götze et al. 1998	Plastic (32), Cadaver (24)	4	IMN, 95°-blade plate	AO 31 A2, A3	Significant higher load to failure with both types of IMN compared with SHS plus TSP
Friedl and Clausen 2001	Plastic (8), Cadaver (2)	5	IMN	AO 31 A2, A3, Subtrochanteric	Higher total and earlier deformation with SHS plus TSP compared with IMN during cyclic loading
Su et al. 2003	Cadaver (10)	10	SHS	AO 31 A3	Significant less sliding distance and displacement of the femoral head in the TSP group
Bong et al. 2004	Cadaver (6)	6	IMN	Evans-Jensen 5	No sign differences in displacement found between the 2 groups during static and cyclic loading
Bonnaire et al. 2007	Cadaver (32)	8	IMN	AO 31 A2.3	Cutout dependant on BMD. All implants sufficient as long as BMD > 0.6 g/cm^3
Walmsley et al. 2016	Composite (24)	NA	IMN	AO 31 A3	Similar stiffness but reduced strength with SHS compared with an IMN.

BMD = bone mineral density

Table 2. Methods of the clinical studies included reporting on the use of sliding hip screw (SHS) with trochanteric support plate (TSP), without comparator (8 studies) or comparing with intramedullary nail (IMN, 6 studies), SHS alone (5 studies), proximal femur locking plate (PFLP, 1 study), Medoff sliding plate (MSP, 1 study) and dynamic condylar screw (DCS, 1 study). In one study an anti-rotation screw (ARS) was used as an addition to SHS

Reference	Design	TSP (n)	Comparator	Fracture classification
Studies without comparator				
Babst et al. 1993	Retrospective cohort	17	None	AO A2,3, A3.3
Hoffmann et al. 1994	Retrospective cohort	19	None	AO A2, A3
David et al. 1996	Prospective cohort	22	None	AO A3
Babst et al. 1998	Retrospective cohort	46	None	AO A2.2, A2.3, A3.3
Gupta et al. 2010	Prospective cohort	46	None	AO
Cho et al. 2011	Retrospective cohort	27	None	AO A2
Prabhakar and Singh 2016	Prospective cohort	25	None	AO A2.1, A2.2, A2.3
Shetty et al. 2016	Prospective cohort	32	None	Evans-Jensen 2–3
Studies with comparator				
Madsen et al. 1998	Retrospective cohort	85	SHS, IMN	Evans-Jensen 3–5
Lunsjö et al. 2001	Prospective cohort	49	MSP, DCS	Evans-Jensen 3–5
Nuber et al. 2003	Retrospective cohort	64	IMN	AO A2.2, A2.3
Klinger et al. 2005	Retrospective cohort	51	IMN	AO A2.3
Hsu et al. 2015	Retrospective cohort	46	SHS	AO A2.1, A2.2, A2.3
Tucker et al. 2018	National database/registry	158	SHS, IMN	AO A2.2, A2.3, A3
Haddon et al. 2019	RCT	50	SHS	Evans-Jensen 3–5
Müller et al. 2019	Retrospective cohort	100	SHS + ARS, IMN	AO A2
Selim et al. 2020	RCT	20	PFLP	AO A2.2, A2.3
Fu et al. 2020	Retrospective cohort	234	IMN	AO A2, A3

Table 3. Results of the clinical studies included reporting on the use of sliding hip screw (SHS) with trochanteric support plate (TSP), without comparator (8 studies) or comparing with intramedullary nail (IMN, 6 studies), SHS alone (5 studies), proximal femur locking plate (PFLP, 1 study), or other extramedullary implants (1 study)

Reference	Clinical outcome	Mechanical outcome	Mechanical failure/non-union (n)	Reoperations n (%)	"Authors' conclusion"
**Studies without comparator**					
Babst et al. 1993	13 patients little or no pain. 10 patients unlimited walking distance.	6 patients 10–25 mm protrusion of lag screw.	0	5 (29)	The TSP prevents excessive lateralization of the greater trochanter
Hoffmann et al. 1994	12 patients walking distance > 200 m. 14 patients little or no pain.	5 patients 10–20 mm protrusion of lag screw. No reoperations at a mean of 6 months follow up. (2 died within 30 d)	0	0 (0)	Low rate of complications and good functional results. More difficult implementation than SHS alone
David et al. 1996	Good functional results dislocation	1 patient with varus	0	0 (0)	TSP recommended in treatment of AO A3 fractures
Babst et al. 1998	Good functional results. 87% excellent/good Salvati Wilson score	Mean impaction 9.5 mm. Mean shortening 6.8 mm.	3	6 (13)	The TSP effectively supports the greater trochanter when the lateral buttress is compromised
Gupta et al. 2010	Good functional results	All fractures healed. 2 TSP removals as buttress is compromised	2	2 (7)	TSP seems to be a useful device for lateral wall reconstruction
Cho et al. 2011	Good functional results with Parker and Palmer mobility score 6.2 (7.2 preop.)	All fractures healed. 1 excessive lag screw sliding and 1 lag screw breakage	0	0 (0)	Additional fixation enables stable fixation of trochanteric fractures and a high rate of union
Prabhakar and Singh 2015	85% excellent/good Harris Hip Score	2 patients with varus collapse and shortening > 2 cm	1	2 (8)	SHS plus TSP is a biomechanically stable construct that allows for lateral wall reconstruction
Shetty et al. 2016	19/32 excellent/good Harris Hip Score	High union rate. Mean RUSH score 21	0	0 (0)	Fixation of unstable trochanteric fractures with SHS plus TSP is an effective technique with good functional and radiological outcome
**Studies comparing SHS/TSP with SHS**					
Hsu et al. 2016	NA	Less lag screw sliding, postop. lateral wall fractures and reoperations with TSP	1	1 (2)	The TSP significantly decreases lag screw sliding distance and reoperation rate in A2 fractures with a critically thin lateral wall compared with SHS alone
Haddon et al. 2019	No difference in functional outcome measured with Merle d'Aubigne score	No difference in radiological outcome or reoperation rates	3	3 (6)	No certain beneficial effect of the TSP on unstable trochanteric fractures compared with SHS alone
**Studies comparing SHS/TSP with other extramedullary implants**					
Lunsjö et al. 2001	No difference in functional outcome	No difference in fixation failure/revisions	3	3 (6)	Extramedullary fixation yields good results with low rate of complications and good functional results. No difference between the examined implants
Selim et al. 2020	Better functional outcome and time to union with SHS plus TSP	Fewer hardware failures and revisions in the SHS plus TSP group	1	1 (5)	SHS plus TSP yields better results than the PFLP in trochanteric fracture treatment
**Studies comparing SHS/TSP with IMN**					
Nuber et al. 2003	Less pain with IMN	Similar complication rates	NA	6 (9)	IMN recommended over SHS plus TSP due to less pain in the IMN group at follow up after 6 months
Klinger et al. 2005	No difference in functional outcome measured with Merle d'Aubigne score	Fewer revisions with IMN. 17% vs 22%	NA	11 (22)	IMN recommended for unstable trochanteric fractures due to more complications in the SHS plus TSP group
Fu et al. 2020 (A2)	No difference in EQ-5D or functional status. More residual pain in the DHS with TSP group	No difference in healing, failure rate or rate of reoperations	10	6 (4)	Good surgical outcome with SHS plus TSP. Comparable results with IMN for both AO A2 on A3 fractures.
Fu et al. 2020 (A3)	No difference in EQ-5D or functional status. More residual pain in the DHS with TSP group	No difference in healing, failure rate or rate of reoperations	2	6 (9)	Good surgical outcome with SHS plus TSP. Comparable results with IMN for both AO A2 on A3 fractures

Table 3. Continued

Reference	Clinical outcome	Mechanical outcome	Mechanical failure/non -union (n)	Reope-rations n (%)	"Authors conclusion"
**Studies comparing SHS/TSP with both SHS and IMN**					
Müller et al. 2019	NA	Better TAD, reduction, and lag screw positioning with IMN. More implant-related complications with SHS ±TSP	11	21 (21)	SHS with TSP associated with more complications and worse radiographical results compared to IMN. IMN recommended for AO A2 fractures
Madsen et al. 1998	Trend towards better functional results with TSP	Less lag screw sliding with TSP. Similar complication rates	5	5 (6)	Fewer associated femoral shaft fractures with TSP compared to IMN and less medialization of the femoral shaft with TSP compared with SHS alone
Tucker et al. 2018	No difference in functional outcome after 12 months. Higher mortality rate with TSP	Similar complication rates	4	4 (3)	IMN conveys the best functional results and fewer revisions when compared with SHS alone or SHS with TSP
